# Haplo-insufficiency of both *BubR1* and *SGO1* accelerates cellular senescence

**DOI:** 10.1186/s13045-016-0238-5

**Published:** 2016-02-04

**Authors:** Sung-Hyun Park, Steve Xie, Chinthalapally V. Rao, Wei Dai

**Affiliations:** Departments of Environmental Medicine, Biochemistry & Molecular Pharmacology, New York University Langone Medical Center, 57 Old Forge Road, Tuxedo, New York 10987 USA; Institute of Pathology, Kings County Hospital Center, Brooklyn, New York USA; Center for Cancer Prevention and Drug Development Program, Department of Medicine, Hem/Onc Section, University of Oklahoma Health Sciences Center, Oklahoma, USA

**Keywords:** *BubR1*, *SGO1*, Knockout mice, MEFs, Senescence

## Abstract

**Background:**

Spindle assembly checkpoint components BubR1 and Sgo1 play a key role in the maintenance of chromosomal instability during cell division. These proteins function to block the anaphase entry until all condensed chromosomes have been attached by the microtubules emanating from both spindle poles. Haplo-insufficiency of either *BubR1* or *SGO1* results in enhanced chromosomal instability and tumor development in the intestine. Recent studies show that spindle checkpoint proteins also have a role in slowing down the ageing process. Therefore, we want to study whether haplo-insufficiency of both *BubR1* and *SGO1* accelerates cellular senescence in mice.

**Methods:**

We took advantage of the availability of *BubR1* and *SGO1* knockout mice and generated primary murine embryonic fibroblasts (MEFs) with mutations in either *BubR1*, *SGO1*, or both and analyzed cellular senescence of the MEFs of various genetic backgrounds.

**Results:**

We observed that *BubR1*^*+/−*^*SGO*^*+/−*^ MEFs had an accelerated cellular senescence characterized by morphological changes and expressed senescence-associated β-galactosidase. In addition, compared with wild-type MEFs or MEFs with a single gene deficiency, *BubR1*^*+/−*^*SGO1*^*+/−*^ MEFs expressed enhanced levels of p21 but not p16.

**Conclusions:**

Taken together, our observations suggest that combined deficiency of *BubR1* and *Sgo1* accelerates cellular senescence.

## Background

Senescent cells are characterized as having gradually lost the ability to divide. Senescence is the main risk factor for age-related disorders including diabetes, atherosclerosis, hypertension, osteoarthritis, and Alzheimer’s disease and Parkinson’s disease [1]. There are two types of senescence including replicative senescence and premature senescence. The replicative senescence is driven largely by telomere shortening [2], whereas the premature senescence is characterized by permanent cell cycle arrest without telomere deterioration [3]. Senescence can be induced by a variety of stressors including UV, oxidative stress, genomic instability, free radical accumulation, and inflammatory cytokines. Senescent cells are characterized by an increased cell size and expression of senescence-associated β-galactosidase (SA-β-Gal). In addition, senescent cells undergo permanent cell cycle arrest, which is accompanied by expressing enhanced levels of negative regulators for cell division including p21, p16, and PTEN.

Spindle checkpoint proteins play a key role in the maintenance of chromosomal instability during cell division [4]. BubR1 is one of spindle checkpoint proteins which functions to block the anaphase entry until all chromosomes have been attached by the microtubules emanating from both spindle poles. Homozygous deletion of *BubR1* results in embryonic lethality [5]. *BubR1* haplo-insufficiency (*BubR1*^*+/−*^) causes enhanced chromosomal instability and tumorigenesis [6]. Extensive research in the past has shown that BubR1-deficient mice also exhibit age-related phenotypes including short lifespan, cachectic dwarfism, lordokyphosis, cataracts, and impaired wound healing [7, 8]. In contrast, increased expression of BubR1 extends healthy lifespan in a mouse model [9]. Therefore, spindle checkpoint protein BubR1 plays an important role in suppressing ageing.

Shugoshin 1 (Sgo1) functions to maintain sister chromatid cohesion, preventing premature cleavage of the cohesin complex by separase [10, 11]. Cells with Sgo1 depletion experience difficulty in completing chromosome congression, leading to accumulation of clusters of chromosomes that surround the spindle poles [12–14]. In the absence of Sgo1, the metaphase plate collapses prior to completion of congression of all chromosome pairs [12]. Missegregation of sister chromatids induced by Sgo1 depletion appears to be responsible for dramatic mitotic arrest [15]. A splice variant of Sgo1 is also involved in the maintenance of centriole cohesion [15, 16]. Similar to many spindle checkpoint proteins, haplo-insufficiency of *SGO1* also predisposes mice to the development of malignancies [10].

Given the availability of *BubR1* and *SGO1* heterozygous mice, we generated primary murine embryonic fibroblasts (MEFs) with mutations in either *BuBR1*, *SGO1*, or both. We studied whether deficiency in the checkpoint gene(s) had an impact on cellular senescence. We observed that *BubR1*^*+/−*^ and *SGO*^*+/−*^ MEFs had an accelerated cellular senescence characterized by the morphological changes and expression of SA-β-Gal. Moreover, double mutant MEFs (*BubR1*^*+/−*^*SGO1*^*+/−*^) exhibited the highest rate of cellular senescence, which was accompanied by early appearance of morphological changes and expression of SA-β-Gal. Compared with wild-type MEFs or MEFs with a single gene deficiency, *BubR1*^*+/−*^*SGO1*^*+/−*^ MEFs expressed elevated levels of p21 but not p16.

## Methods

### Mouse colonies and MEFs

Transgenic knockout mice (*BubR1*^*+/−*^ mice and *SGO1*^*+/−*^ mice) were maintained at the animal facility of New York University Langone Medical Center. The maintenance was fully compliant with the general guidelines outlined by the United States Department of Agriculture and the American Association of Laboratory Animal Care under the supervision and specific approval of the Institutional Animal Care and Use Committees. *BubR1*^*+/−*^*SGO1*^*+/−*^ compound mutant mice were obtained by crossing *BubR1*^*+/−*^ mice with *SGO1*^*+/−*^ mice. Primary MEFs of various genotypes were obtained from embryos (embryonic day 14.5) of mice of *BubR1*^*+/−*^ and *SGO1*^*+/−*^ crossing. MEF cells were cultured at 37 °C under 5 % CO_2_ in 100-mm plates containing Dulbecco minimum essential medium (DMEM) supplemented with 10 % fetal bovine serum (FBS) and antibiotics (100 μg/ml of penicillin and 50 μg/ml of streptomycin sulfate; Invitrogen). Serial cultures were carried out in which cells were trypsinized and replated at a density of 10^6^ cells/100-mm dish every 7 days.

### Genotyping

Genotyping was carried out as previously described [5, 10]. Briefly, genomic DNA was purified from mouse tail or MEFs. The PCR for detecting BubR1 genotype was carried out with PCR primers as follows: the forward primer A of 5′GGG AGG ATC GAG GAG GTC G3′, the forward primer long-terminal repeat 2 (LTR2) of 5′AAA TGG CGT TAC TTA AGC TAG CTT GC3′, and the reverse primer B of 5′CTG TTC GCC TTC AGT GCT CAA AAT GGT AGT CG3′. The reaction conditions included an initial denaturation at 94 °C for 5 min followed by 35 cycles of 94 °C for 30 s, 58 °C for 30 s, and 72 °C for 1 min. The final extension of the reactions was set at 72 °C for 7 min. The PCR for *SGO1* genotyping was carried out with primers as follows: the forward primer of 5′GAA AAG TAA GTC TGC TTA TGG CTC A3′, the reverse primer of 5′CAG GTG TTG TAG AAT AAT CCA AGC3′, and the reverse primer long-terminal repeat (LTR) of 5′ATA AAC CCT CTT GCA GTT GCA TC3′. The reaction conditions included an initial denaturation at 94 °C for 3 min followed by 35 cycles of 94 °C for 45 s, 57 °C for 45 s, and 72 °C for 1 min. The final extension was set at 72 °C for 10 min. PCR products were analyzed by electrophoresis on 2 % agarose gels and detected by ethidium bromide staining.

### Senescence-associated β-galactosidase staining

MEF cells were seeded at a density of 3 × 10^4^ cells (at passages 3 and 4), 4 × 10^4^ cells (at passage 5), 5 × 10^4^ cells (at passage 6), 6 × 10^4^ cells (at passage 7), and 7 × 10^4^ cells (at passage 8) per well, respectively, on 12-well plates. One day after seeding, MEFs were stained for SA-β-Gal activity according to the manufacturer’s protocol (Cell Signaling Technology). The percentage of senescent cells was the total number of senescent cells divided by the total number of cells counted under a light microscope.

### Western blot analysis

Western blot analysis was carried out as previously described [17, 18]. Total MEF cell lysates were prepared in the RIPA lysis buffer (50 mM Tris-HCl (pH 8.0), 150 mM NaCl, 1 % NP-40, 0.1 % SDS, and 0.5 % sodium deoxycholate) supplemented with a mixture of protease and phosphatase inhibitors. Protein concentrations were measured using the bicinchoninic acid protein assay (Pierce™ BCA Protein Assay Kit, ThermoFisher Scientific). Equal amounts of protein lysates from various samples were used for SDS–PAGE analysis followed by immunoblotting. Luminata Forte Western HRP substrate (Millipore) was used for chemiluminescent detection.

### Statistical analysis

Statistical analysis and graphs were generated using the Prism 6 (Graphpad). Data were analyzed for significant differences using the two-tailed, independent *t* test with Welch’s correction for un-equal variances, or with the Mann-Whitney *U* test. A *p* value <0.05 was considered statistically significant.

## Results and discussion

**Fig. 1:**
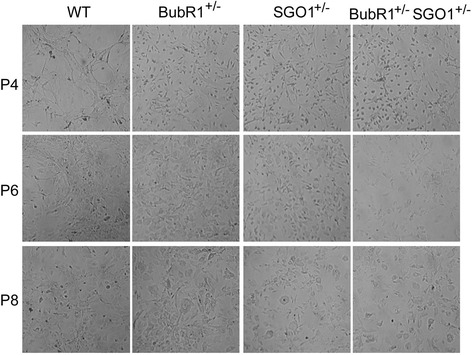
*BubR1* ^*+/−*^ *SGO1* ^*+/−*^ MEFs exhibit senescent-like morphologies at an accelerated rate. Primary MEFs of various genetic backgrounds (wild-type, *BubR1* ^*+/−*^, *SGO1* ^*+/−*^, and *BubR1* ^*+/−*^ *SGO1* ^*+/−*^) were cultured in DMEM supplemented with FBS and antibiotics as described in the Methods section. Serial cultures were carried out at a density of 10^6^ cells/100-mm dish every 7 days. At each passage, images of MEFs were recorded with a light microscope. Images of MEFs of wild-type (*WT*), *BubR1* ^*+/−*^, *SGO1* ^*+/−*^, and *BubR1* ^*+/−*^ *SGO1* ^*+/−*^ at passages 4, 6, and 8 are shown

It is well documented that deficiency in spindle checkpoint control results in chromosomal instability, aneuploidy, and tumorigenesis [19–21]. Recent studies have revealed that aneuploidy due to a weakened spindle checkpoint accelerates ageing [7]. For example, mice carrying hypomorphic *BubR1* alleles develop a series of ageing-related pathologies that include shortened life span, growth retardation, fat loss, and impaired wound healing [7]. Given that *SGO1* also plays a role in the maintenance of chromosomal stability during cell division and that it remains unclear whether *SGO1*-deficiency also contributes to the accelerated ageing process, we first obtained primary MEFs that were either haplo-insufficiency of *BubR1*, *SGO1*, or both. Morphological examination revealed that *SGO1*-deficient MEFs appeared to be similar to that of *BubR1*-deficient ones, which grew at a slightly faster rate than the wild-type MEFs (data not shown). However, compound mutant MEFs (*BubR1*^*+/−*^*SGO1*^*+/−*^) grew at a much slower rate, and a small fraction of these cells exhibited morphologies of senescent cells at early passages (e.g., P4) (Fig. 1). Many MEFs became large, flat, and/or multinucleated. With each passage, these morphologically distinct cells significantly increased in *BubR1*^*+/−*^*SGO1*^*+/−*^ MEFs compared with wild-type MEFs. By passage 8, a high fraction of *BubR1*^*+/−*^*SGO1*^*+/−*^ displayed senescent cell morphologies. These senescent-like cells were also present in *BubR1*- and *SGO1*-deficient cells at late passage (passage 8).

**Fig. 2:**
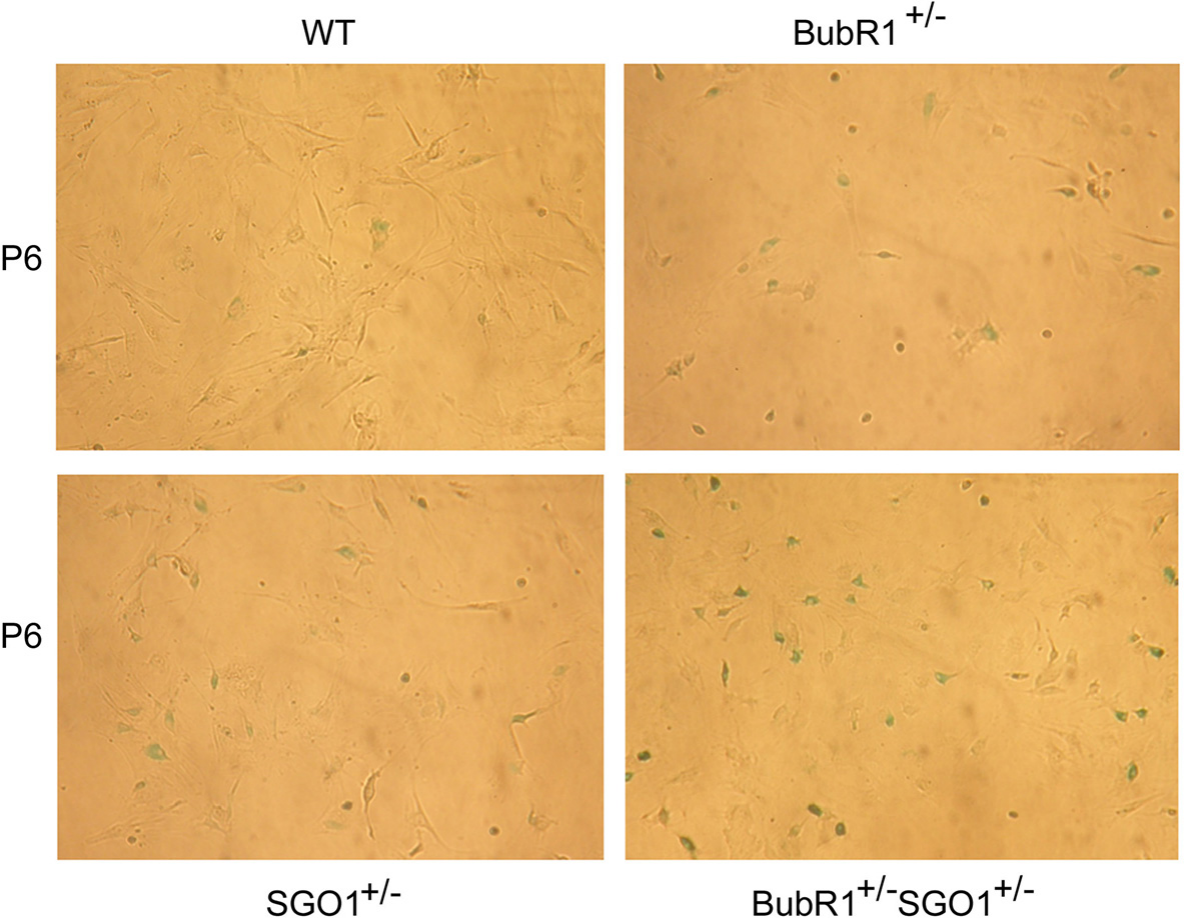
*BubR1* ^*+/−*^ or *SGO1* ^*+/−*^ deficiency leads to enhanced expression of β-galactosidase. Primary MEFs of various genetic backgrounds (wild-type, *BubR1* ^*+/−*^, *SGO1* ^*+/−*^, and *BubR1* ^*+/−*^ *SGO1* ^*+/−*^) at passage 6 were stained for senescence-associated β-Gal activity (blue according to the manufacturer’s protocol. β-Gal-positive cells were stained with blue

**Fig. 3:**
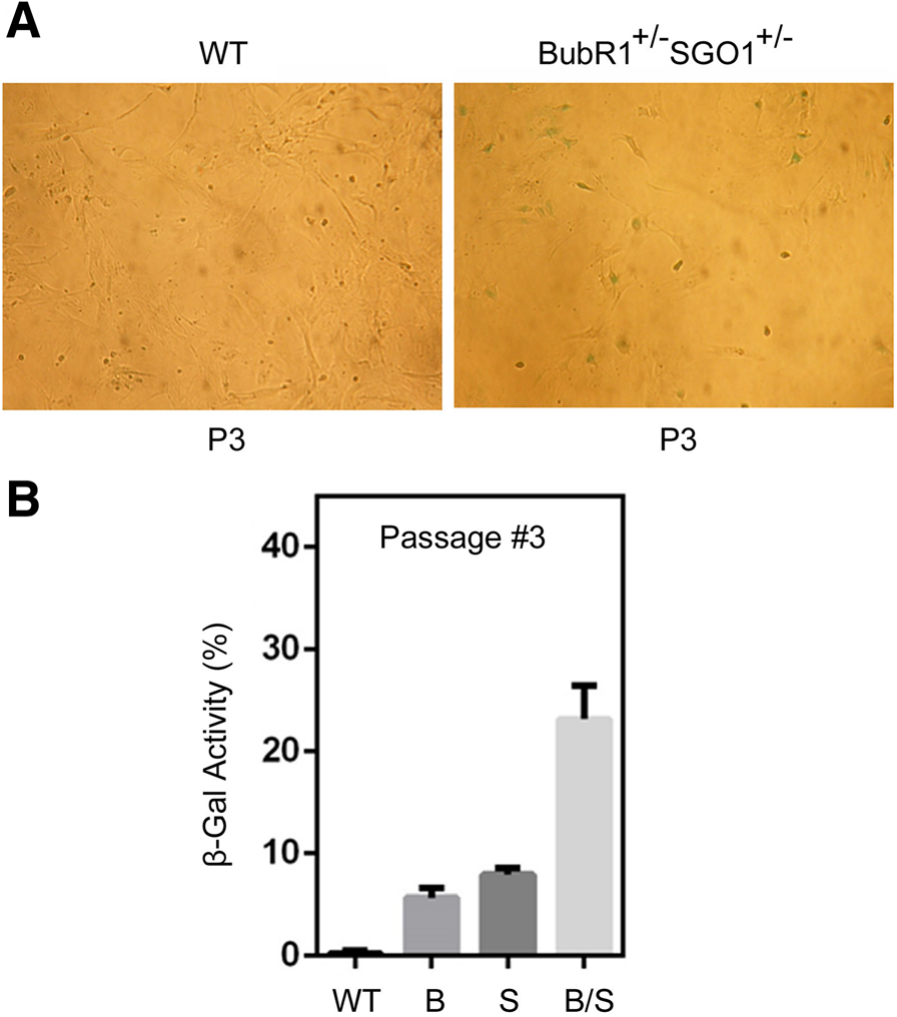
*BubR1* ^*+/−*^ *SGO1* ^*+/−*^ MEFs express SA-β-Gal much earlier than wild-type *BubR1* ^*+/−*^ or *SGO1* ^*+/−*^ MEFs. **a** Primary MEFs of various genetic backgrounds (wild-type and *BubR1* ^*+/−*^ *SGO1* ^*+/−*^) at passage 3 were stained for senescence-associated β-Gal activity. **b** Primary MEFs of various genetic backgrounds (wild-type *BubR1* ^*+/−*^, *SGO1* ^*+/−*^, and *BubR1* ^*+/−*^ *SGO1* ^*+/−*^) at passage 3 were stained for senescence-associated β-Gal activity. β-Gal-positive cells were recorded. Summarized data from three independent experiments are shown

Given that lysosomal SA-β-Gal is a reliable and widely used biomarker for senescent cells, we stained MEFs of various genotypes for SA-β-Gal expression. We observed that a significant number of *BubR1*^*+/−*^*SGO1*^*+/−*^ MEFs at very early passages (e.g., passage 3) already expressed SA-β-Gal where few, if any, wild-type MEFs expressed this enzyme (Fig. 2). Some MEFs with a single gene deficiency (*BubR1*^*+/−*^ or *SGO1*^*+/−*^) also expressed SA-β-Gal, indicative of their senescent status. However, MEFs with compound mutations displayed a much higher tendency toward senescence. At passage 6, a significant number of *BubR1*^*+/−*^ or *SGO1*^*+/−*^ MEFs also expressed SA-β-Gal (Fig. 3). However, *BubR1*^*+/−*^*SGO1*^*+/−*^ MEFs expressed SA-β-Gal at a higher level, evidenced by the stronger signals of SA-β-Gal than MEFs with a single gene deficiency.

**Fig. 4:**
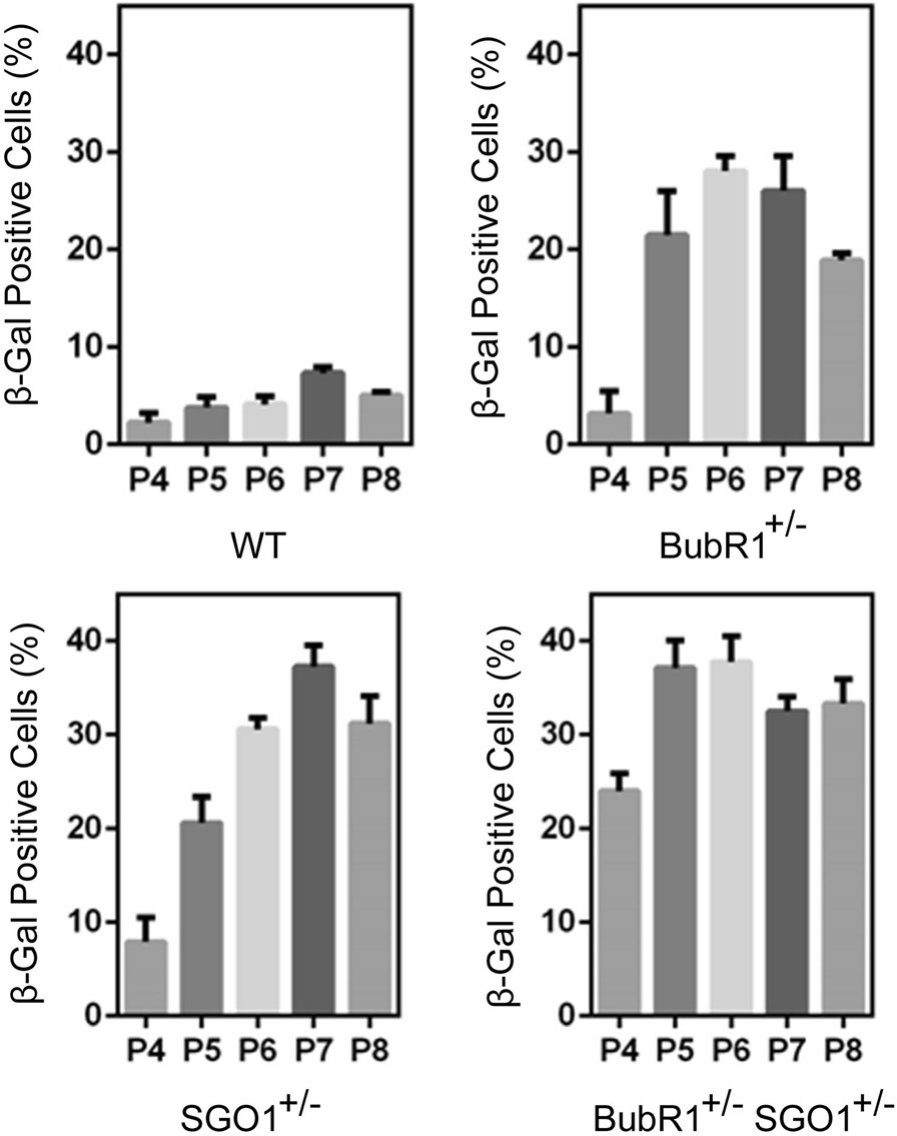
*BubR1* ^*+/−*^ *SGO1* ^*+/−*^ compound mutations accelerate senescence. Primary MEFs of various genetic backgrounds (wild-type, *BubR1* ^*+/−*^, *SGO1* ^*+/−*^, and *BubR1* ^*+/−*^ *SGO1* ^*+/−*^) were cultured in DMEM as described in the Methods section. MEFs of different passages were stained for SA-β-Gal. Percents of SA-β-Gal cells were recorded. Data summarized from three independent experiments are presented

We then systematically analyzed SA-β-Gal expression in MEFs of various genotypes at different passages. We observed that a small fraction (around 3 %) of wild-type MEFs started to express SA-β-Gal at passage 4, and there was gradual increase in the percentage of SA-β-Gal-positive cells (Fig. 4). On the other hand, *BubR1*^*+/−*^ MEFs exhibited a significant increase of SA-β-Gal-positive cells around passage 5, reaching the peak level at passage 6. Slightly different from *BubR1*^*+/−*^ MEFs, *SGO1*^*+/−*^ MEFs showed a significant increase in the percentage of SA-β-Gal-positive cells at passage 4, reaching the peak level at passage 7. The percentage of SA-β-Gal-positive cells in *SGO1*^*+/−*^ MEFs was higher than that in *BubR1*^*+/−*^ MEFs. Moreover, in addition to early (passage 3) display of cellular senescence, *BubR1*^*+/−*^*SGO1*^*+/−*^ MEFs also had a higher percentage of cells positive for SA-β-Gal at passages 4 and 5. It appears that the highest percentage of SA-β-Gal-positive cells in all genotypes was around 37 %. Thus, there is a synergy of premature ageing between reduced expression of *BubR1* and *SGO1*.

**Fig. 5:**
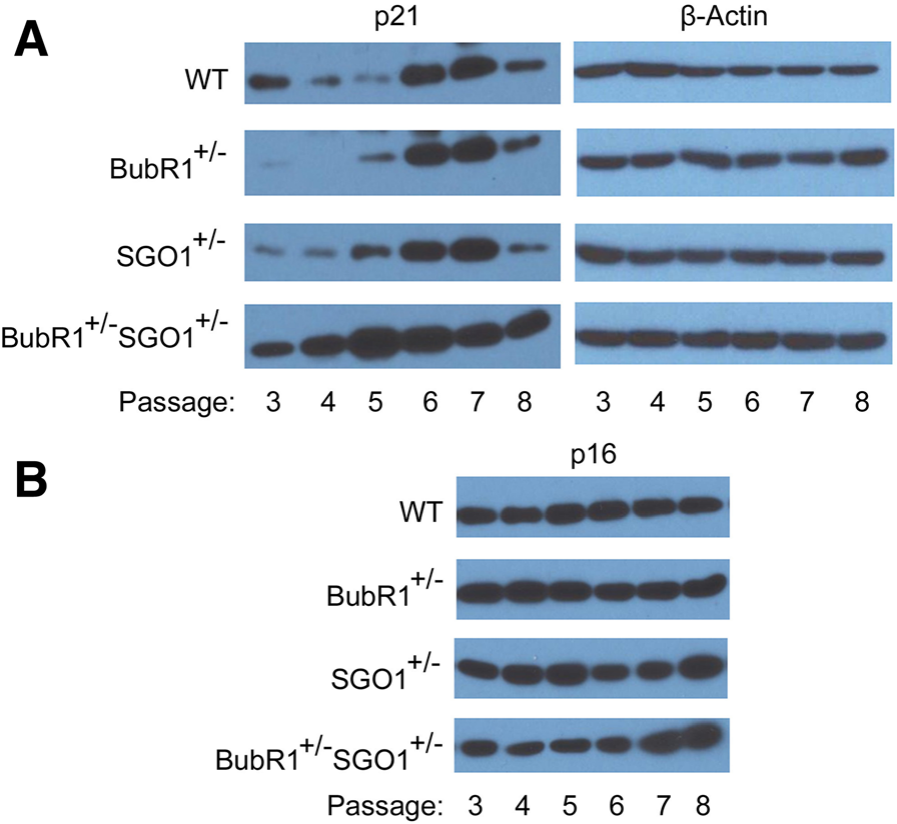
**a** *BubR1* ^*+/−*^ *SGO1* ^*+/−*^ MEFs express enhanced levels of p21. MEFs of various genetic backgrounds (wildtype, *BubR1* ^*+/−*^, *SGO1* ^*+/−*^, and *BubR1* ^*+/−*^ *SGO1* ^*+/−*^) were cultured and passed in vitro as described in the Methods section. MEFs of different passages were collected and lysed. Equal amounts of cell lysates were blotted for p21 and β-actin. **b** The same set of lysates were blotted with antibody to p16

To further confirm accelerated ageing due to the reduced expression of spindle checkpoint genes, we analyzed several molecular markers including p21 and p16 that are frequently linked to cellular senescence. At passage 3, *BubR1*^*+/−*^*SGO1*^*+/−*^ MEFs had a high level of p21 expression compared with that of *BubR1*^*+/−*^ or *SGO1*^*+/−*^ MEFs (Fig. 5). Expression of p21 further increased with additional passages, which was roughly correlated with percentages of SA-β-Gal-positive cells. On the other hand, there was little difference of p16 expression among early passages until passages 7 and 8 in *BubR1*^*+/−*^*SGO1*^*+/−*^ MEFs. Moreover, there was no significant difference of p16 expression between *BubR1* (or *SGO1*)-deficient and *BubR1* (or *SGO1*)-competent MEFs. These observations suggest that p16 expression may not be responsible for the observed cellular senescence in MEFs that are haplo-insufficiency of *BubR1*, *SGO1*, or both.

In this study, we systematically analyzed cellular senescence in cells deficient in BubR1 and/or Sgo1. It is known that reduced expression of BubR1 induces premature ageing which is associated with enhanced cellular senescence [7]. We have shown for the first time that haplo-insufficiency of *SGO1* also causes increased senescence. Sgo1 appears to play a more important role in suppressing ageing as haplo-insufficiency of *SGO1* induces cellular senescence earlier and to a greater extent than haplo-insufficiency of *BubR1*. At the molecular level, BubR1 and Sgo1 function differently in suppressing premature separation of sister chromatids [20]. BubR1 is an integral component of the spindle checkpoint by suppressing the activity of APC/C [22, 23], whereas Sgo1 is physically associated with cohesin, preventing its premature cleavage by separase. Therefore, it is reasonable to predict that there is a synergy between BubR1 and Sgo1 in suppressing premature sister chromatid separation and chromosomal instability in cell division. Consistent with this prediction, we have observed that reduced expression of both BubR1 and Sgo1 results in accelerated cellular senescence.

We have shown that enhanced senescence is correlated with elevated expression of p21 but not p16. As it is well known that cellular senescence is associated with permanent cell cycle arrest, it is logical that expression of CDK inhibitors such as p21 is significantly elevated. Although we did not observe that p16 expression is high in MEFs that are undergoing senescence, we cannot exclude the possibility that expression of other inhibitors of the INK family including p15, p17, or p19 is elevated. Although they all function to block cell cycle progression, p21 and p16 act differently in the negative regulation of CDKs. As a member of the INK family, p16 solely acts as an inhibitor that blocks G1 CDKs (CDK4/6). On the other hand, p21 is capable of inhibiting CDKs of several cell cycle transitions including S and G2/M. Given that BubR1 and Sgo1 have primary functions in mitosis, it is logical to speculate that impaired functions of these checkpoint components can trigger cell cycle arrest and senescence by activating p21 expression.

## Conclusions

This study is consistent with the notion that chromosomal instability due to reduced expression of spindle assembly checkpoint components contributes to ageing processes. The combined deficiency of *BubR1* and *SGO1* accelerates cellular senescence which is manifested as morphological changes and enhanced expression of SA-β-Gal.
